# Dengue Detection: Advances in Diagnostic Tools from Conventional Technology to Point of Care

**DOI:** 10.3390/bios11070206

**Published:** 2021-06-23

**Authors:** Md Alamgir Kabir, Hussein Zilouchian, Muhammad Awais Younas, Waseem Asghar

**Affiliations:** 1 Asghar-Lab, Micro and Nanotechnology in Medicine, College of Engineering and Computer Science, Boca Raton, FL 33431, USA; mkabir2016@fau.edu (M.A.K.); hzilouchi@knights.ucf.edu (H.Z.); 2 Department of Computer & Electrical Engineering and Computer Science, Florida Atlantic University, Boca Raton, FL 33431, USA; 3 College of Medicine, University of Central Florida, Orlando, FL 32827, USA; 4 Services Institute of Medical Sciences, Lahore 54000, Pakistan; aawaisyounas@hotmail.com; 5 Department of Biological Sciences (Courtesy Appointment), Florida Atlantic University, Boca Raton, FL 33431, USA

**Keywords:** dengue, diagnostics, point-of-care

## Abstract

The dengue virus (DENV) is a vector-borne flavivirus that infects around 390 million individuals each year with 2.5 billion being in danger. Having access to testing is paramount in preventing future infections and receiving adequate treatment. Currently, there are numerous conventional methods for DENV testing, such as NS1 based antigen testing, IgM/IgG antibody testing, and Polymerase Chain Reaction (PCR). In addition, novel methods are emerging that can cut both cost and time. Such methods can be effective in rural and low-income areas throughout the world. In this paper, we discuss the structural evolution of the virus followed by a comprehensive review of current dengue detection strategies and methods that are being developed or commercialized. We also discuss the state of art biosensing technologies, evaluated their performance and outline strategies to address challenges posed by the disease. Further, we outline future guidelines for the improved usage of diagnostic tools during recurrence or future outbreaks of DENV.

## 1. Introduction

The DENV is a flavivirus that is primarily spread by the female *Aedes aegyptus* mosquito, which lives mostly in an urban environment [1]. The first virologic proved case of dengue in the USA was in Philadelphia in 1780 [2]. However, the first dengue like symptoms in the Americas were reported in 1635 [2]. Once a non-infected mosquito bites an infected human, the incubation period lasts for 4–10 days inside mosquito [3]. After the incubation period, the vector can transmit the virus for the rest of its life.

**Figure 1:**
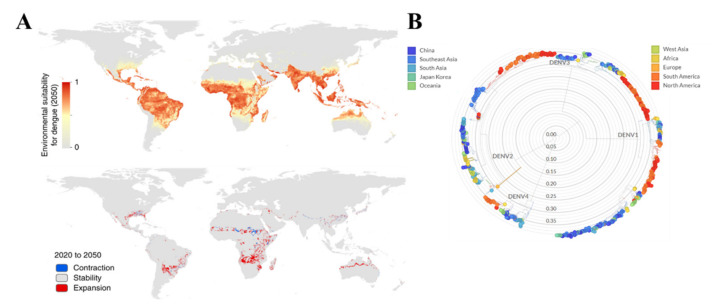
(**A**) Worldwide estimated distribution of dengue in next 30 years due to the climatic and population change [8]. (**B**) Phylogeny analysis of 922 complete genomes evolution of all DENV serotypes reported from January 2000 to October 2020 based on their origin. (Source: Nextstrain.org (accessed on 15 May 2020)).

An estimated 390 million persons are infected yearly, with 2.5 billion people being in danger of contagion in most subtropical and tropical areas [4,5]. The DENV from 1960 to 2010 has seen a 30-fold upsurge due to an increase in global population, global warming, ineffective mosquito control, and inadequate medical facilities [6]. Every year, around 100 million people become sick, requiring medical attention and around 22,000 people die due to the dengue virus infection globally [7]. The DENV affects more than 100 countries worldwide, including first-world countries such as the USA [6]. In the next 30 years, Dengue is estimated to expand more due to change in population density, urbanization and climatological conditions (Figure 1A) [8]. However, currently, 70% of the endangered countries are Asian, and a large number of infections are still being recorded in countries like Bangladesh, Malaysia, Vietnam, and the Philippines [3]. Besides, 2,163,354 dengue cases of infections has been reported in Americas in 2020 with 872 deaths [9].

DENV can be distinguished into four serotypes which can be related both genetically and antigenically as follows DENV-1, 2, 3, and 4 [10,11]. All the four DENV distinct serotypes are evolving over the subtropical areas in Asia, Africa, Europe, North and South America (Figure 1B). The DENV typically causes a flu-like illness that can affect all ages, but also more-severe disease manifestations are also common, including plasma leakage [12,13]. Some typical infection signs are a high fever running around 104°F, aches behind the eyes, severe headache, muscle/joint pain, vomiting, enflamed glands, and rash [14]. These symptoms can last up to 7 days, but they appear 4–10 days after the mosquito bites the person. However, severe dengue fever (DF) can be potentially life-threatening in part due to plasma leaking, fluid accumulation, ascites, pleural effusions, severe bleeding, low platelets, and/or organ impairment [14]. Nevertheless, patients infected with DENV-2 & DENV-4 shows acute illness due to dengue hemorrhagic fever (DHF), but infection due to DENV-1 & DENV-3 is mild, sometimes inapparent [15]. DHF can be staged in four grades according to the guidelines of World Health Organization (WHO): Grade I- only mild bruising, grade II- spontaneous blood loss into the skin and in another place, grade III- a symptoms of shock, and grade IV- acute shock [3,16]. Currently, there is no gold standard antiviral treatment for DF/DHF; however the maintenance of a patient’s body fluids plays a crucial role in the treatment [17]. To date, only one vaccine (Dengvaxia, Sanofi Pasteur, Marcy-l’Étoile, France) has been licensed for use in several countries but can only be administered to people with a previous infection [18].

Diagnostic testing varies in type, cost, and time for the DENV. Nucleic acid amplification tests (NAATs) and serologic tests are both used to detect the virus [19]. The viral load measurement by detecting genomic nucleic acids in infected patients is considered the gold standard for diagnosing dengue infection at the preliminary stage of infection [20]. Alternatively, the NS1 protein in the DENV holds clinical significance by allowing individuals to detect the virus in the early phase (0 to 14 days) [21]. The immune response against the viral infection starts to develop after a few days of symptom onset. As a result, IgM and IgG response from the immune system is also considered a possible medium of dengue virus diagnostic [20]. Besides, the DENV infection cocirculates with other flaviviruses, especially with Zika virus; thus, specific detection of DENV plays a vital role in DENV outbreak management [22]. Conventional diagnosis methods require sophisticated medical facilities as well as trained personnel. Most of the developed tests are not suitable for resource limited settings although DENV is distributed mostly in countries with limited resources. False positive/negative results further restrain the implementation of dengue control measures as only one third of the diagnosed cases are confirmed by another testing [23]. As a result, there is an unmet need for the development of highly specific point of care (POC) diagnosis platforms suitable for resource constrained areas following the WHO Affordable, Sensitive, Specific, User-friendly, Rapid and robust, Equipment-free and Deliverable to end-users (ASSURED) criteria [24]. Various state of the art biosensing technologies have recently been developed for the low-cost and rapid detection of DENV utilizing fluorescence, colorimetric or impedimetric principle of detection. The use of these biosensor-based assays requires lesser amount of sample. High sensitivity and specificity, and portability makes these biosensors a suitable alternative to conventional lab-based assays for early stage of detection. There are also reports of multiplexed assays which can play significant role in detecting and differentiating DENV from other flaviviruses where DENV is endemic.

In this review, we discuss the current conventional technologies utilized for testing DENV at resource available settings. We also discuss, analyze and compare their performances with the methods that are being developed or recently commercialized. Moreover, we also discuss the currently developed state of the art biosensing technologies, evaluated their performance and outline strategies to address challenges posed by disease and provide future guidelines for the improved usage of diagnostics during recurrence or future outbreaks of DENV. This review is comprehensive covering both laboratory-based conventional and POC diagnostic methods and bridging the information gaps that were not discussed in other reviews published recently [25,26,27,28,29,30,31,32]. We focus mainly on the most recent advancements in POC based biosensing assays that were developed since 2015. We also highlight recent advancements in dengue sensing, including CRISPR-based methods, which were not covered previously.

## 2. Dengue Structure

**Figure 2:**
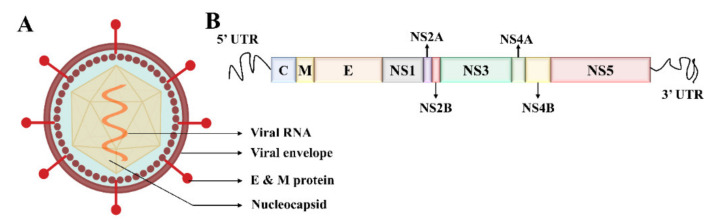
Structure of the dengue virus and evolution. (**A**) Dengue virus is spherical and about 40–60 nm in size. Genomic RNA and nucleocapsid proteins are covered by a membrane called Envelope. (**B**) The complete dengue virus genome is ~11 kb long, consisting of three structured and seven non-structured proteins and two untranslated regions (not drawn to the scale).

Electron microscopy imaging demonstrates that DENV has a relatively smooth surface, 40–60 nm size, and contains 25–30 nm nucleocapsid protein covered with a lipid bilayer (Figure 2A) [33,34]. The DENV is a positive-sense ssRNA virus that can be translated directly to proteins [1]. The complete genome is ~11 kb-long, encoding for three structural and seven nonstructural proteins, and the subtypes of DENV share about 65% of the complete amino acid sequences (Figure 2B) [1,35]. E, C, and prM/M are the structural proteins, while the nonstructural (NS) proteins are named NS1, NS2A, NS2B, NS3, NS4A, NS4B, and NS5 [35,36]. DENV also contains two untranslated 5′ and 3′ terminal regions in the genome [37,38]. The virus is structured with Envelope/E protein with 495 amino acids and consists of three domains that mainly interact with the host cell for invasion [39].

## 3. Conventional Methods of Laboratory Diagnostics

### 3.1. Serological Tests

Serological tests are most widely used to detect dengue infection due to low cost and operative simplicity compared to molecular or culture-based testing. Serum and cerebrospinal fluid specimens are most commonly used for testing the presence of IgM and neutralizing antibodies. However, plasma and whole blood samples are used occasionally as testing specimens. The presence of the IgG antibody can also work as a detection biomarker for dengue. IgG level persists in the human body after primary infection. It can exhibit inconclusive results for secondary infection and false positive in those with infection/immunization against other flaviviruses (West Nile virus (WNV), yellow fever virus (YFV), or Zika virus (ZIKV)).

#### 3.1.1. IgM-Based Tests

**Figure 3:**
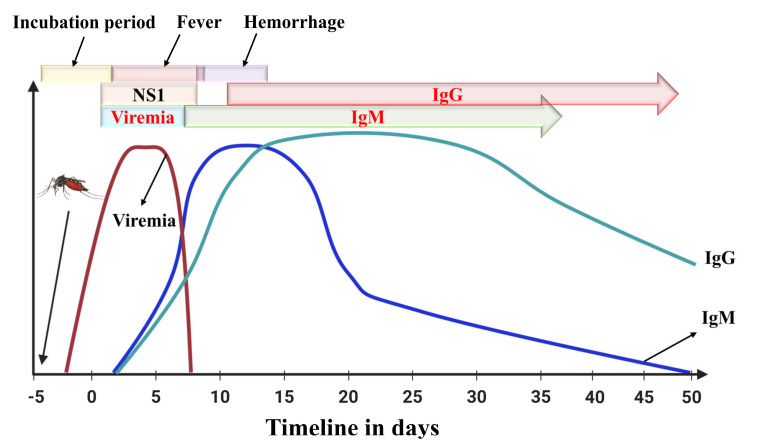
Immune response by the human body against the first invasion of the Dengue virus.

Antibodies are proteins generated by the immune system to fight against the attack of an antigen [40]. Immunoglobulin proteins, for example Ig(G, M, A, E, and D) are conventional antibodies along with B-cells and T-cells are produced in response to antigens. During an infection, IgM antibodies are usually produced five days after the onset of symptoms, and they persist in the human patient’s body for 2–3 months, sometimes even longer [1]. For devices that detect Dengue antibodies, the major problem is that samples are collected before the five-day negative window period (Figure 3). First-time dengue infections have a higher IgM antibody response, while secondary infections have a higher IgG antibody response [41]. An enzyme-linked immunosorbent assay capturing the IgM antibody (MAC-ELISA) test, first developed by the Armed Forces Research Institute of Medical Sciences, can be utilized to sense IgM antibodies in dengue [42].

There are numerous commercially available MAC-ELISA testing kits that are developed and marketed for testing dengue infection globally. To perform the test, usually, the dengue IgM antibody is captured from the patient’s serum specimen by anti-human IgM antibodies on a solid phase. A sandwich-type immunoassay is then developed using recombinant dengue-derived antigen and DENV-specific monoclonal antibody [43,44,45]. In the United States, the Food and Drug Administration (FDA) has given clearance to the first such test named DENV Detecta IgM Capture ELISA by InBios International, Inc. (Seattle, WA, USA) [46,47]. It provides qualitative detection by capturing the human IgM antibody to DENV recombinant antigens on a 96 well plate [48]. To verify the positive test a Plaque Reduction Neutralization Test (PRNT) is done [49]. To clinically prove the effectiveness of InBios test, the FDA conducted five clinical studies in different sites. The first clinical study showed that 2–3 days of onset fever and 4–5 days of onset fever showed only a 28.7% (2–3 days) and 40.3% (4–5) chance of a positive result. However, after 6–7 days, the percentage went up to 75.9% of a positive result, and after 8–10 days, the percentage went up to 88.7% [45,50]. Another study tested the cross-reactivity of the assays. The only virus that was cross-reactive was the WNV [51]. This assay presented a sensitivity of 96% and a specificity of 94% [50].

There are several inherent drawbacks to these IgM rapid tests and IgM-based ELISA tests. The low sensitivity of the POC tests can be a considerable drawback in clinics. In the United States, only one such POC test is approved, and before six days, the test is unreliable to a certain extent. Due to some cross-reactivity, a PRNT based test is needed to confirm the results in some cases.

The incubation period for dengue fever is usually five days, and the acute illness can be observed from 0–7 days of disease infection, and sometime after that period, some patients experience hemorrhagic shock. During the early stage of infection, viral load measurement can be done using RT-PCR or any other molecular detection method. Simultaneously, NS1 can also be diagnosed up to 10 days of infection by ELISA method. IgM or IgG based testing is useful to diagnose dengue infection after a few days of infection.

#### 3.1.2. IgG-Based Tests

IgG-based tests can be used for determining past or present infections. The IgG antibody is produced after IgM, and IgG antibodies can persist for a more extended period (Figure 3), even lifelong [52]. A four-fold spike in IgG antibodies is often attributed to a recent infection [53]. Standard Diagnostics (Seoul, South Korea) and Panbio Inc (Brisbane, Australia now Alere Inc., Waltham, WA, USA) have commercialized IgG-based ELISA tests [54].

The Standard Diagnostics IgG-based ELISA has a sensitivity of 81.2% and specificity of 39.8%. Panbio, on the other hand, had a sensitivity of 63.5 and specificity of 95.3% [54]. Other IgG ELISA tests from companies such as Inbios (Seattle, WA, USA), Abcam (Cambridge, MA, USA) and Euroimmum (Lübeck, Germany) also exist [55]. A study was done comparing these tests with their NS1 and IgM/IgG antibodies and observed that the IgG tests had a high rate of false positives and high cross-reactivity [55]. IgG tests can be useful in certain instances, but IgM and NS1 ELISA are more useful for acute infections. Due to cross-reactivity with other flavivirus IgG antibodies, it is challenging to determine primary infection just by testing IgG-based assays [56].

#### 3.1.3. IgM/IgG Ratio Tests

An IgM/IgG ratio test is usually differentiating test between first time and later infections [57]. Optical density (OD) is measured in both IgM and IgG ELISA tests. A ratio greater than 1.32 is considered as primary infection, while a ratio below 1.32 is considered secondary [58]. Specific laboratories often regulate the cutoff value of the OD ratio to determine the outcome of the infection. One laboratory in Northern India determined that the best cutoff ratio is 1.1 [59]. The use of both IgM and IgG can be useful to determine the type of infection present. Depending on the IgM and IgG test performed, the cutoff ratio will vary.

#### 3.1.4. Hemagglutination Inhibition Test

The hemagglutination Inhibition (HI) test is a standard test for distinctive primary and later DENV infections, but it cannot detect early infection [60,61]. Red blood cells or tympanized human O red blood cells agglutinates due to the presence of viral antigen [62]. Anti-dengue antibodies can prevent agglutination, and the robustness of the inhibition is tested by this test [63]. Response to a primary infection usually has low levels of antibodies, while secondary infections have high antibody titers that usually exceed 1:1280. Values below 1:1280 are usually interpreted as a primary infection [63]. A study was done where an IgG ELISA and HI were tested to show if they can differentiate between primary and secondary infections [64]. IgG ELISA divided sample OD with a mean of calibrator OD. Any value below 1 was characterized as a primary infection, and any amount over 1 was a secondary infection [64]. The study found that HI and IgG were able to detect primary infection ideally with 16/16 samples detected. However, for secondary infections HI was inferior to the IgG-based test. HI test was only able to detect 23/73 secondary infections, while the IgG ELISA test was able to identify 72/73 samples [64]. The IgG ELISA test is much easier to perform than HI testing, and IgG testing is much more reliable. HI also fails to distinguish between other flaviviruses [62]. However, HI tests are easier to be completed than plaque reduction neutralization test (PRNT) because the viral culture is not required for HI [65].

#### 3.1.5. Plaque Reduction Neutralization Test

A PRNT allows an individual to determine exactly the source of infection in IgM positive individuals by detecting precise neutralizing antibodies. PRNT assay was first developed in 1967 by Russel and Nisalak [66,67]. PRNT is also considered the gold standard method to test the WHO’s vaccine immunogenicity [68]. The PRNT test is usually used in a particular instance where serological information is needed or to confirm a case. The test is generally done in a test tube or a microtiter plate [69]. A specific antibody has the ability to neutralize the virus. When the virus is neutralized, plaques cannot form, so in theory, if the antibody inactivates the virus, then plaques do not form [66]. To start the test, cells are coated with semi-solid media on a test tube or plate that does not allow the spread of the progeny virus. The serum specimen is serially diluted and being mixed with a standard amount of the virus [46]. In the next few days, plaques will form and can be counted through dying so plaques can be observed. Then the plaques are compared to determine the amount decrease entire virus infectivity to the concentration of the virus [70].

The PRNT can be done at the Centers for Disease Control and Prevention (CDC, Atlanta, GA, USA) or a lab nominated by CDC. However, worldwide, there is no set standard for PRNT assay [70]. Without a set standard, comparing the results with PRNT can be cumbersome and can alter the interpretations of results [67]. PRNT is also used to effectively determine asymptomatic DENV infections, although it showed cross-reactivity with other DENV serotypes [71]. A noteworthy drawback of this test is that it is time-consuming and labor-intensive.

#### 3.1.6. NS1 Based Tests

Nonstructural 1 (NS1) antigen plays significant part in replicating DENV into the host cell. The antigen is produced and discharged into the bloodstream of the infected patients; as a result, it is considered as an essential biomarker for detecting flavivirus infection at an earlier stage [72,73,74]. NS1 tests are particularly important in clinical settings because they can detect the acute phase of the DENV, and NS1 persists longer than viremia in blood [19,75,76,77,78]. Usually, lateral flow-based rapid assays are used as rapid detection schemes, and antigen-capture ELISAs are used in laboratory-based testing [79]. According to CDC, the NS1 based tests show similar outcomes as molecular tests in the first week of infection [67]. There is currently one FDA approved NS1 test in the USA, the DENV Detect NS1 ELISA (InBios International), and it provides qualitative results [80]. However, currently, there are seven commercially available tests, with four of them being rapid tests [81,82]. The seven tests are Dengue NS1 Ag STRIP (BioRad, Marnes-la-Coquette, France), Platelia Dengue NS1 Ag ELISA (BioRad), Dengue NS1 Detect Rapid Test (InBios International), DENV Detect NS1 ELISA (InBios International), Panbio Dengue Early Rapid (Alere, Waltham, WA, USA), Panbio Dengue Early ELISA (2nd generation (Alere), and SD Bioline Dengue NS1 Ag Rapid Test (Abbott, Abbott Park, IL, USA) [81,83].

Several studies have been reported to evaluate the sensitivity and specificity performance of all these testing devices. However, the Detect NS1 ELISA (InBios International), which is the only FDA approved product in the market, has a sensitivity of 95.9%, which makes it the superior product among its peers (89.4% by BioRad and 85.6% by Panbio) [84]. The sensitivity varied by DENV stereotype with DNEV-1 reporting the highest sensitivity. DENV-4 sensitivity had more variation, but the Detect NS1 ELISA test by InBios International had a 100% sensitivity with DENV-4. The Biorad and Panbio tests, on the other hand, have a sensitivity of 75 and 66.6 percent, respectively. However, different studies show different specificities and sensitivities, more or less, closer to the studies mentioned above [23,55,81,83,85,86,87,88].

Rapid tests can be done in a resource-limited settings where access to adequate health care facilities might not be available. All the rapid tests usually take less than 30 min to perform. However, all the rapid tests showed a statistically significant decrease in sensitivity compared to the FDA-approved Detect NS1 ELISA by InBios International. The highest sensitivity for the rapid tests was for NS1 Ag STRIP by BioRad, which showed a sensitivity of 81.0 to 84.8 percent for all serotypes and a specificity of 100% [89]. The InBios and SD Bioline Dengue NS1 Ag rapid test presented 76.5% and 72.4% sensitivity, respectively [81]. The Inbios rapid test had a lower specificity of 97.3% compared to SD rapid test, which had a specificity of 100%. Finally, Panbio showed a 71.9% sensitivity and 95% specificity, performing the worst among its peers [47]. For all the tests, the lowest sensitivity was for DENV-4, while the highest was for DENV-1.

A positive NS1 confirms a DENV infection, while a negative result does not eliminate infection possibility. If a negative test result occurs, then an IgM based test should be performed [67]. A notable downside of NS1-based tests is that they are not recommended after seven days [90].

### 3.2. Molecular Detection

Nucleic acid tests (NATs) are molecular tests done in a centralized lab location and require expensive equipment and skilled personnel. Most NATs require RNA/DNA extraction, which in most cases, cannot be performed in POC settings. NATs diagnose and quantify viral RNA/DNA with higher accuracy and sensitivity, allowing detection in the acute phase. The viral genome can be detected within five days of symptom onset. Several assays have already been developed that may exhibit quantitative detection or provide semi-quantitative or qualitative detection.

#### 3.2.1. Polymerase Chain Reaction (PCR) Based Tests

The most common DENV NAT test is PCR which is also considered as the gold standard for detecting DENV at an earlier stage of infection due to its higher sensitivity [91]. In RT-PCR, the viral RNA is initially extracted from different samples, including plasma, blood, urine, and serum. Then viral RNA is transcripted to cDNA, followed by amplification, and the fluorescence from the amplified RNA is read by devices to determine positive and negative results [92]. However, most of the recently developed RT-PCR based platforms usually target all the serotypes and can differentiate each of them. Recently, a multiplex RT-PCR assay was developed to determine serotypes from the blood sample, and it can be used during blood transfusion [93]. The developed assay was tested against all serotypes of DENV and was successful in detecting as low as 100 viral copies/mL. Another single step RT-PCR based assay was developed where primers were designed such that they can detect and differentiate DENV from ZIKV, YFV, and Chikungunya virus [94]. This assay is highly sensitive and showed no cross-reactivity. However, the conventional PCR requires skilled personnel and lab facilities to perform this manual assay. To automatize the sample extraction to amplification, an insulated isothermal PCR assay has been developed to detect DENV [95]. They developed POCKIT combo central system which accommodates a cartridge where serum samples are loaded and the cartridge contains all the extraction, amplification reagent and the POCKIT system automatically provides the qualitative result with a lowest detection points of 1 and 10 PFU/mL for DENV-1,3 and DENV-2,4 respectively.

There are currently two NAT assays approved by the FDA: CDC DENV-1-4 Real-Time RT-PCR Multiplex assay and Trioplex rRT-PCR Assay [96]. The CDC DENV-1-4 Real-Time RT-PCR Multiplex assay is used in instances when the primary cause of infection would be the DENV. The assay differentiates DENV sreotypes [96]. The Trioplex rRT-PCR Assays are used when Dengue, Chikungunya, and Zika are apparent [97]. This allows a laboratory to test for all three viruses simultaneously. The FDA did a study to show the effectiveness of the CDC DENV RT-PCR Multiplex Assay [98]. The observed LOD in serum and plasma was reported as 1 × 10^3^ PFU/mL. The FDA conducted a prospective and retrospective study. The prospective study showed a 97.2% positive agreement while a 100% negative agreement. In the retrospective study, the FDA indicated that it could detect RNA in 98.04% of the samples and 98.5 in negative percent agreement [98]. Furthermore, a study compared the CDC assay and a laboratory-built assay [99]. This study showed that the lab assay had a higher sensitivity of 97.4% compared to 87.1% of the CDC assay.

The Trioplex can differentiate between the DENV, ZIKV and Chikungunya viruses [100]. However, it cannot distinguish between the dengue serotypes. The Trioplex is only usable for symptomatic patients, not for blood donors. The test can detect Dengue in serum, cerebrospinal fluid, and blood but not in urine. The LOD for the DENV in this complex ranges from 6 to 15  copies/reaction [101]. The test showed no cross-reactivity with other flaviviruses [102]. It has 100% positive and negative agreement for both DENV and Chikungunya viruses. In comparison, ZIKV differed slightly for both positive and negative percent agreement showing 95% and 99.1 % agreement, respectively [100].

#### 3.2.2. Isothermal Amplification Based Tests

Another form of molecular diagnostics is isothermal amplification of genomic DENV RNA, where the amplification process requires only one temperature. In rural areas, for example, access to lab-based PCR tests might not be possible. Isothermal amplification-based tests do not require a thermocycler, so the cost and the complexity compared to PCR can be reduced [103]. Unfortunately, to date, there is no CDC approved isothermal product for the detection of DENV. Although, several platforms are already being developed that utilized isothermal amplification method, including reverse transcriptase loop-mediated isothermal application (RT-LAMP), Reverse Transcription Recombinase Polymerase Amplification (RT-RPA) and nucleic acid sequence-based amplification (NASBA).

**Figure 4:**
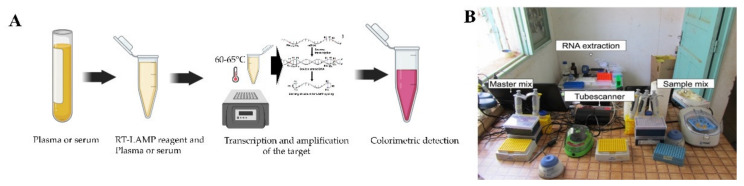
Detection of DENV RNA using isothermal amplification based platforms. (**A**) Detection of DENV RNA using LAMP method, initially RNA is transcripted to dsDNA and then amplified by the primers creating a loop structure (Modified from [107]). (**B**) A mobile laboratory to be used at point of care to detect DENV using RPA method utilizing minimal reagents as well as equipment (reprinted from [124]).

The RT-LAMP assay, first developed by Notomi in 2000, consists of four to six primers: two outer primes, two inner primers, and two loop primers recognizing eight distinct regions on the target [104,105]. This method has been developed as a promising pathogen detection technique that uses only water bath or a simple heating block to amplify the viral target at a constant temperature, usually in the range of 60–65 °C [106]. Usually, the RT-LAMP starts transcripting the target to DNA (Figure 4A), followed by creating a loop structure that creates copies of the target. The amplified target can be visualized by the naked eye or sometimes under UV light [107]. A study done by S. hu et al. showed the effectiveness of RT-LAMP [108]. The RT-LAMP assay they designed showed a 100% and 98.9% success rate for diagnosing clinical strains of DENV and infected patients, respectively. In their study, the RT-PCR had a sensitivity of 93% for clinical strains and 84.2% for infected patients. No false positives were found with the RT-LAMP. Targeting the C-prM gene of all DENV serotypes, Li et al. have recently reported a single tube reaction method utilizing the RT-LAMP primers within 30 min [109]. Lau et al. also published a single tube RT-LAMP method focusing 3′-NCR gene of all DENV serotypes and detected the DENV serum by analyzing the change in turbidity, with LOD of 10 RNA copies per reaction [110]. In a recent report, DENV RNA is amplified using LAMP and then integrated with a portable MinION sequencer to perform serotyping [111]. RT-LAMP has many advantages; such as it is rapid, cost-effective, isothermal, highly sensitive and specific. However, in the case of multiplexing and viral quantification applications, PCR is superior to LAMP-based assays [112]. LAMP-based assay usually provides qualitative (Yes/No) results, without any quantification data.

Another commonly used isothermal amplification method is NASBA. The main advantage of this assay is that it is a single-step isothermal process targeting the RNA of the DENV samples [112]. Silica is used to extract the RNA from the clinical serum or plasma samples, which is then amplified without a thermocycler at 41°C [113]. The amplification can be done within 30 min and then could be detected by electrochemiluminescence [114]. Several conventional NASBA-based DENV detection assays have been reported recently [115,116,117,118]. Yrad et al. reported the DENV-1 RNA detection platform using AuNPs [119]. DENV-1 target RNA is amplified by NASBA amplification followed by generation of sandwich complex by AuNP probe and DENV-1 capture probe. This complex can be visually detected with a lateral flow biosensor within 20 min in pooled human sera with a LOD of 1.2 × 10^4^ pfu/mL. Although NASBA is simple, cost-effective, and it increases the detection sensitivity of the biosensor used but it requires sample pre-processing and cautious handling of the viral targets [120].

RPA assay is another novel isothermal amplification technique that has been developed for detecting pathogens [121]. RPA is able to detect 1–10 DNA copies of target per reaction within 20 min at a constant temperature in the range of 37–42 °C [122]. Teoh et al. developed an RPA based DENV detection assay targeting highly conserved 3′-UTR regions [123]. The LOD of this assay was 10 copies of DENV RNA per reaction and it provided results within less than 20 min. A point of need based mobile RPA unit (Figure 4B) has been developed and deployed in Thailand and Senegal to ensure sensitive detection of DENV rapidly [124]. The team developed two assays targeting DENV 1-3, and DENV 4 to cover all the serotypes by targeting the 3′-UTR regions. The mobile unit facilitates necessary equipment and reagents for RNA extraction to fluorescent detection. Moreover, another RT-RPA based assay was developed where the detection was done by lateral flow dipsticks with a detection capability of 1 to 10^6^ copies/μL for DENV-1 [125]. Considering all the abovementioned RPA based assays it is clear that this platform requires minimally expensive equipment and can be employed in resource constraint settings for high and accurate detection of DENV.

## 4. Recently Emerged State-of-the-Art Sensing Technologies for POC DENV Detection

Currently, there is a need for POC technologies that can be cheaper and reliable in various health care settings. This emerging market has many technologies that have the potential to be used in these settings. Technologies such as electrochemical impedance spectroscopy (EIS) based sensing, surface plasmon resonance (SPR), microarray-based sensing, paper-based lateral flow assays, and lab-on-chip (LOC) are currently being researched to become POC technologies so they can be clinically used in these settings. Nanoparticles are often combined with these technologies to enhance performance. The FDA has not cleared any of these products for clinical use for DENV; however, they have fundamental features to be considered for POC applications. Here we focus on the state of art sensing technologies that have been just developed or are currently being developed.

### 4.1. Surface Plasmon Resonance Based Technologies

**Figure 5:**
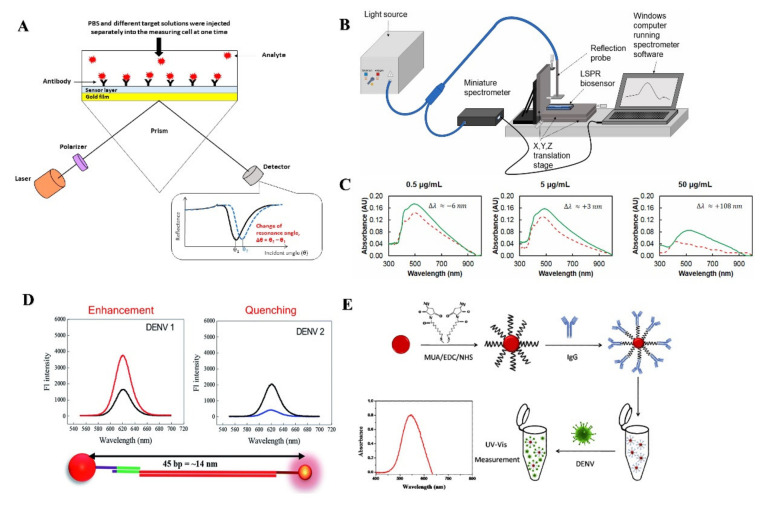
Detection of DENV viral biomarkers using surface plasmon resonance technologies. (**A**) An Au thin film based graphene oxide-polyamidoamine dendrimer based SPR sensor mechanism represents to measure the change in the SPR angle in present of DENV viral proteins (Reprinted from [128].). (**B**,**C**) Silver nanostructured SPR biosensor experimental setup to detect different concentration of NS1 antigen by measuring the peak wavelength shift on the chamber of a nanostructured device. NS1 antigen (green solid line), and anti-NS1 antibody (red dashed line) (Reprinted with permission from ref. [130]. Copyright 2019 Elsevier.). (**D**) Detection of different DENV serotypes based on altered distance based fluorescence signal (LSPR Enhancement and quenching) generated by primer–probe conjugated quantum dots and AuNPs (Reprinted from [133]). (**E**) AuNPs surface was modified by a self-assembled monolayer of MUA which facilitated covalent bonding of the DENV specific antibody with the AuNPs. (Reprinted with permission from ref. [134]. Copyright 2018 Elsevier.).

Surface plasmon resonance (SPR) a real-time detection method that uses polarized light to sense the variations in a refractive index (RI) that occur in the vicinity of a sensor surface [27]. The first known use of SPR was in 1902 to investigate optical properties, and later it has been used in numerous biosensing applications [126]. SPR detects changes in the RI when a biomolecular interaction between antigen and antibody occurs [127]. When the reaction occurs, the light hitting the surface has a changed spectrum due to angular shift (Figure 5A) [128]. SPR has many advantages that include the fact that they are label-free, high sensitivity, and low sample consumption [129]. In recent times, several SPR based sensors are developed to detect NS1 antigen, IgM antibody as well as IgG antibody. One recently developed SPR based sensing device can detect NS1 antigen of DENV in human plasma samples within 30 min [130]. A silver nanostructure was created using thermal annealing method, which enabled the blood plasma separation using a polyethersulfone membrane in a microfluidic device. The DENV NS1 antigen was immobilized by an anti-NS1 antibody on the chamber of the nanostructure surface to create a peak wavelength shift based on the antigen-antibody binding (Figure 5B,C). This sensor relates higher the antigen-antibody binding with higher peak wavelength shift. In another report, an Au SPR chip fabricated with a self-assembled monolayer developed by the use of 11-mercaptoundecanoic acid has shown improved detection ability than a conventional IgM capture antibody ELISA to detect IgM antibodies with 10–100% dilution range [131]. The presence of IgM antibodies in the serum was correlated with an increase in resonance angle [129]. The technique is called the rapid IgM-based dengue diagnostic test, which has the potential to use the four DENV serotypes as a ligand [132]. However, the SPR test they developed was not able to differentiate between the serotypes. This developed assay has 83–93% sensitivity and 100% specificity in human serum samples.

Another group of researchers developed a LSPR-based sensing mechanism using serotype-specific nanoprobe bound to CdSeTeS quantum dots and Au nanoparticles to detect different serotypes of DENV [133]. The distance dependent fluorescence signal was generated based on LSPR enhancement and quenching, which also determined the serotypes for DENV (Figure 5D). Another easier to operate approach for detecting DENV serotypes using surface modified AuNPs based LSPR sensor was reported recently (Figure 5E) [134]. The 22±5 nm AuNPs surface was modified by 11-mercaptoundecanoid acid, and then antibody IgG antibody was conjugated covalently to the AuNPs surface to capture DENV antigen. This method of DENV detection reduced the time of detection of DENV to less than 5 min. SPR based biosensors were also used to detect the E protein of DENV at a very low (0.08 pM) concentration [135]. SPR possesses features to be used in POC settings due to its high sensitivity, real time response and low sample consumption. However, the lower sensitivity of SPR based assays compared to some IgM tests could pose some issues for health care providers. Though, the POC potential is there due to the fact that the test only requires very low (one microliter) volume of patient serum [132]. So far, no SPR technology has been approved by the FDA nor CDC. A disadvantage of SPR is that it is susceptible to nonspecific binding and suitable only for convalesce stage. This can hamper its ability to detect the DENV in some settings.

### 4.2. Electrochemical Sensing-Based Technologies

**Figure 6:**
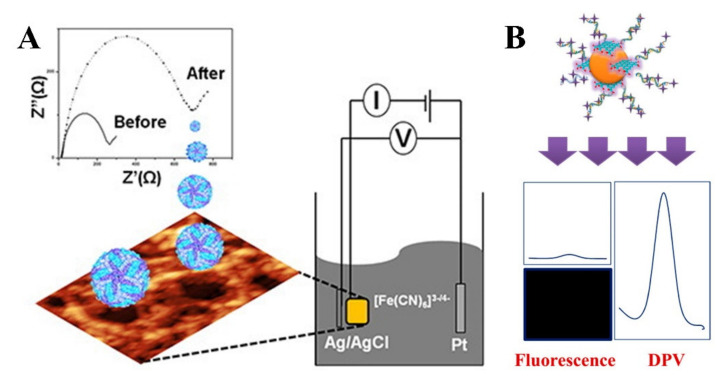
Electrochemical detection of DENV. (**A**) Electrochemical impedance sensing technique for DENV detection using graphene oxide and polymers (Reprinted with permission from ref. [154]. Copyright 2017 Elsevier.). (**B**) DENV virus detection using differential pulse voltammetric method utilizing a combination of nano composites (Reprinted with permission from ref. [160]. Copyright 2018 American Chemical Society.

Electrochemical sensing (ES) can measure changes in electrical properties (e.g., impedance, voltage, and current) from the biomolecular interactions on the surface of different biosensors, which has been used for detecting various viral targets [103,136,137,138,139,140]. Electrochemical impedance spectroscopy (EIS) also has the ability to keep a sample intact with minimal damage [141]. EIS can measure a vast array of frequencies, which allows it to detect these electron transfers, chemical reactions, and electrolyte conductance due to biological interactions [142,143,144]. Recently, advancements have been made to detect the DENV using EIS [145,146,147]. EIS possesses applicability in POC settings for early stage DENV detection due to its cost-effectiveness, high throughput, and high sensitivity. Moreover, EIS offers label free detection of specific antigen-antibody reactions [145]. Till now, different electrochemical based biosensor development approaches have been reported, including carbon nanotube, screen printing, gold nanoparticles, and peptides [148,149,150,151]. EIS technology based on gold nanoparticles (AuNPs) was developed for DENV 1-4 [145]. In this assay, a gold electrode was coated with cysteine. Instead of DNA primers, this AuNPs device monitored the signal response for antibody binding. There is also progress in utilizing EIS technologies involving graphene for RNA and DNA detection of DENV [141]. Graphene is an attractive material for electrical applications due to its electrical conduction, chemical stability, and mechanical strength [152]. This can lead to higher sensitivity, low detection limits, and long-term stabilities for biosensors [153]. Navakul et al. created a biosensor by taking graphene oxide (GO) with polymer matrix composites coated on the gold electrode surface to be used to detect DENV using EIS (Figure 6A) [154]. To measure the resistance change on the biosensor the impedance signal was measured with DENV and DENV with 4G2 DENV antibody conjugate. The higher signal reduction was observed in the case of DENV and 4G2 DENV antibody, confirming the binding of DENV on the electrode. The lower LOD achieved with this method was 0.12 pfu/m [154]. Carbon nanotubes (CNTs) have also been incorporated into EIS technology to detect the DENV through the NS1 antigen and whole virus [155,156]. Palomar et al. immobilized DENV-2 NS1via polypyrrole-NHS activated amide coupling [157]. The carbon nanotube layer contains the immobilization reaction of DENV-2 NS1. Their device has a sensitivity of 45.7 ± 1.7 Ω/order of the magnitude of antibody concentration change in PBS. The LOD of this CNT based assay was 10–12 g/mL [157].

Another commonly used analytical sensing technique is voltammetric sensing. Voltammetric sensing can be either cyclic voltammetry (CV) or differential pulse voltammetry (DPV). CV is used to analyze the redox potential and the electrochemical rates by measuring the resulting current change with respect to applied varying potential [32]. On the other hand, DPV measures the before and after application of linear increment of amplitude potential pulses to analyze the redox process [158]. The advantages of the voltammetric sensing are cost effectiveness, rapidness, and the low requirement of reagents. Recently, various voltammetric sensing assays have been reported for detecting DENV; for example, Palomar et al. developed an electrochemical sensor with Au and carbon nanotube based composites [159]. Both CV and DPV-based analysis strategies have been adopted to detect the DENV 2 NS1 antigen in this assay. DENV virus molecular detection is also possible with this method. A composite of Au and nitrogen, sulfur co-doped graphene quantum dots has been used to detect DENV DNA using four dye-combined probes with a LOD of 9.4 femto molar [160]. In this report, DPV technique was applied to detect and quantify the DENV with the presence of methylene blue (Figure 6B) and a fluorescence tag was used to determine the serotype of DENV. Another voltammetric electrochemical sensing assay known as square wave voltammetry (SWV) has also been reported recently to detect DENV NS1 antigen [161].

The development of electrochemical sensing based techniques has become a powerful tool for DENV detection in POC application for their diverse and multitude advantages. In general, this method proved to be superior than some other techniques for their label free and higher antigen antibody affinity characteristics. Besides, the detection sensitivity can be improved by using different types of nanostructures. However, some chemical sensor development requires use of clean room facilities which increases the cost of the sensor. Further, significant sample pre-processing is required before electrochemical methods can be used.

### 4.3. Surface-Enhanced Raman Spectroscopy Based Technologies

Surface-enhanced Raman spectroscopy (SERS) is a sensing mechanism that was discovered in 1974 [162]. SERS is a method that enhances Raman spectroscopy by improving the electron cloud around metallic nanostructures, which is done through either electromagnetic or chemical enhancement [163]. Electromagnetic enhancement is done through LSPR wavelengths in resonance with the Raman source, while the chemical enhancement occurs through a molecule bound directly to the metal surface. It was recently showed that SERS active enhancement factors could achieve 106 to 108 M. Furthermore, a 2D silence on Ag (111) showed an enhancement factor to 109 [164]. It provides a chemical fingerprint on an object to ensure higher specificity, requiring a less complex sample preparation mechanism, free from interference of signals capability, high reproductivity and potential to be implemented in POC settings. SERS can be used for various areas such as various viral pathogen diagnostics, including HIV, Influenza virus, Respiratory viruses, SARS-CoV-2 and flaviviruses [165,166,167,168,169,170].

Recently SERS has been adapted to detect infectious diseases such as Zika and DENV [170]. The idea to detect DENV is to use the NS1 antigen. The DENV-4 serotype can be detected using a SERS active Ag-Au bimetallic nanowire chip. The ssDNA is immobilized onto the Au, and the signals were measured without washing the sample. With SERS, there was no need to use reagents, and there was no need to mark the ssDNA for the detection of DENV. SERS based detection provided LOD of 7.67 ng/mL [170]. This low LOD shows the promising use of SERS to detect infectious diseases like DENV. Furthermore, a SERS assay with a cascade amplification strategy was developed by C. Song et al., and they reported LOD as low as 0.49 fM [171]. ]. This would allow clinicians to diagnose DENV in the early stages of the disease and improve the prognosis. The testing for clinical samples using a SERS platform based on Ag nanorod array was carried out in a study [172]. The team of investigators was successfully able to differentiate infected and non-infected patients based on the recorded SERS spectra.

SERS currently holds promise as a POC in the future, but it still inherently lacks robustness and repeatability compared to the current gold standard assays for DENV. A considerable limitation of SERS is the high initial cost of portable Raman readers that might not be available in many rural and developing areas [164]. However, SERS shows promise in detecting DENV at early onset as it provides low LOD. SERS can also lower the cost of testing over the long term due to the fact that it does not require a washing step where reagents are used.

### 4.4. Microfluidics-Based Sensing Technologies

**Figure 7:**
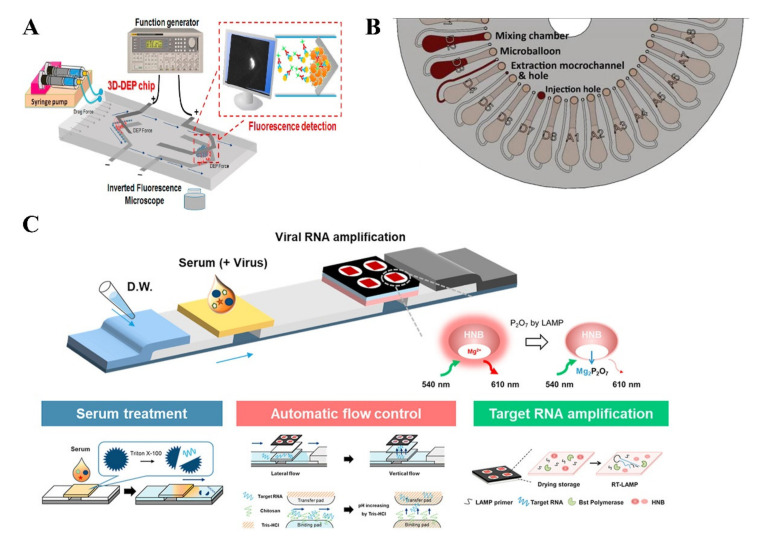
Detection of DENV using microfluidic-based sensing technologies. (**A**) Experimental setup of a microfluidic chip based DENV detection using dielectrophoresis technique. AC voltage was supplied by a waveform generator and inverted microscope was used to record the fluorescence of the captured virus (Reprinted with permission from ref. [179]. Copyright 2017 Elsevier.) (**B**) A multilayered and PMMA made lab on a disc microfluidic device to ensure ultrasensitive detection of DENV (Reprinted from [185]). (**C**) Lab on a paper device to detect multiple pathogen targets accommodating amplification of RNA using LAMP method (Reprinted with permission from ref. [191]. Copyright 2020 Elsevier.).

Microfluidic-based sensing encompasses a wide variety of tests such as lab on a chip (LOC), lab on a disc (LODc), lateral flow assays (LFA), and microfluidic paper-based devices (μPADs) [128,173]. LOC embodies numerous steps into one platform, which allows real-time detection while only using a small amount of the sample [174]. These steps include biochemical reactions, transportation, product detection, and sample/ analyte concentrators [175]. There are several LOC technologies that have been developed to detect various viral targets, as well as devices that incorporate micro/nanoparticles [135,176,177,178]. Often microfluidic-based sensing incorporates paper material and nanotechnology together to enhance detection. LOC can detect a wide variety, such as IgM/IgG antibodies, NS1 antigens, E protein, and RNA. LOC-based devices can be fabricated using both photolithographic and non-photolithographic techniques. A photolithographic three-dimensional microfluidic chip has been developed to detect DENV (Figure 7A) using the dielectrophoresis method [179]. To ensure rapid detection, 4G2-coated beads were used and coupled with a fluorescent probe to ensure fluorescence detection. The developed chip is highly reusable and requires a very small sample volume, which reduced the cost of detection per assay. Another microfluidic assay has been developed recently to detect DENV RNA from whole blood with the help of microbeads, where DENV RNA is amplified using the LAMP method [180]. The full “sample-in and answer-out” process takes less than one hour to complete, and it can detect as low as 10^2^ PFU per 200 μL. LOC-based DENV sensing is faster than conventional PCR and ELISA testing and provides feasibility to be used at resource-limited settings. LOC can also be fully automated and less expensive than other traditional tests, which gives it tremendous potential to be used in a POC settings to detect the DENV [181]. Moreover, LOC-based assays provide manipulation of bioparticle and reagents based on their dimensions and forms [179].

On the other hand, LODc is another microfluidic device that utilizes centrifugal force to pump the reagents in different chambers inside a disc [182]. It also requires a minimal amount of reagent and is cost-effective due to non-photolithographic fabrication techniques. It also facilitates different types of detection capabilities [183]. A lab on a compact disc (LOCD) based DENV IgG antibody detection platform has been reported [184]. They have used motor controlled rotation to facilitate specific movement of the disc to ensure the antigen-antibody reaction. A photodetector measures the color changes in OD in the last chamber of the disc and sends the OD value to a smartphone via Bluetooth connection [184]. The platform has 95.2% sensitivity and 100% specificity in clinical samples. Moreover, a multilayer PMMA based LODc has been developed where microspheres and microfluidic disc formed a hybrid that is used to detect DENV (Figure 7B) [185]. A sandwich ELISA-based detection technique has been adopted by microballoon mixing mechanism inside the disc. The system is fully automated and requires 5 min to precisely detect 1.9 pfu/mL DENV in serum. The main advantage of this LODc based devices is that they don’t require expensive material to fabricate and also have the option to automatize the assay [186].

LFA’s are paper-based tests that are comprised of a nitrocellulose membrane where antibodies are immobilized by electrostatic interactions [187]. LFA’s are based on the liquid’s movement through the capillary force and antibody-antigen binding complex [174]. A secondary antibody is introduced that, if captured, causes a color change (line formation) that is seen on test [174]. Currently, there are numerous LFA devices on the market for DENV detection targeting the NS1 and IgM. A paper microfluidic hybrid chip was developed that integrates LFA for NS1 detection [188]. Yuzon et al. developed a 2.4 cm × 2.4 cm chip fabricated using nitrocellulose membrane, polymethyl methacrylate, and cellulose acetate film. The researchers compared the chip to the gold standard method of a sandwich ELISA. The sandwich ELISA takes 24 h to complete and provides a sensitivity of 43.932 ng/mL. However, their chip has a LOD of 84.66 ng/mL having 2 min readout time. The device’s sensitivity is clinically relevant as DENV infected patient’s plasma can have 0.04–2 μg/mL of NS1 in the first week of infection [189]. The chip also requires less sample volume and a more straightforward disposal process [188]. Zhang et al. developed a lateral flow based assay to detect DENV target antibody (IgM and IgA) from a large volume of saliva samples using a simple stacking flow based 2D platform [190]. The simple 2D paper based stacking device is assembled in such a way that ensures a separate flow path for saliva and reagents. On the other hand, a 3D vertical and lateral flow lab-on-paper device has been developed to target multiple viruses, including ZIKV, DENV, and chikungunya virus (Figure 7C) [191]. This device accommodates the whole LAMP-based NAT process from the dried reagents by completing serum treatment, automated flow control, and finally, the RNA amplification. The amplification takes place at 65 °C and the whole process takes less than 60 min to complete. Ortega et al. has developed an ELISA using magnetic polydopamine-based nanoparticles to detect the IgM antibodies of DENV [192]. This device is highly sensitive and can detect as low as 40 ng/mL of DENV IgM. Recently, another DENV NS1 based detection platform has been developed, which can detect as low as 5 ng/mL by capture-layer lateral flow immunoassay [193]. Moreover, micro size paper-based analytical devices commonly known as μPADs have been incorporated with smartphones to detect the DENV [177,194]. μPADs consist of a paper that is hydrophilic in nature and various polymers that can have hydrophilic interactions [177]. Cellphones are often utilized to collect data. The data can then be interpreted at a central lab or at point of use. Prabowo et al. created a μPAD that utilizes a sandwich immunoassay on paper having wax pattern to detect the NS1 antigen [195]. The total test time was around 25–30 min. They found the LOD of the assay by naked eye, a cellphone camera and scanner with values of 200, 74.8 and 46.7 ng/mL, respectively, indicating that smartphones can be incorporated in a testing setup. Theillet et al. have developed a laser cut μPAD to detect DENV NS1 antigen from the pediatric serum sample [196]. A microfluidic pump was developed to control the flow inside the chambers of the device. Microfluidic lateral flow based testing can also be fully automated and less expensive than other traditional tests. However, there are some shortcomings. The sample preparations of these tests often take a lot of time and skilled personnel to perform manual step by step sample loading [187]. This can make it not viable in some rural areas where POC testing is needed.

### 4.5. Non-Conventional Microfluidic-Based Sensing Technologgies

There are some other microfluidic-based technologies that are being used for DENV detection with a potentiality to be used at POC. In this section, we discuss microarray and thread based assays for their application in DENV detection.

**Figure 8:**
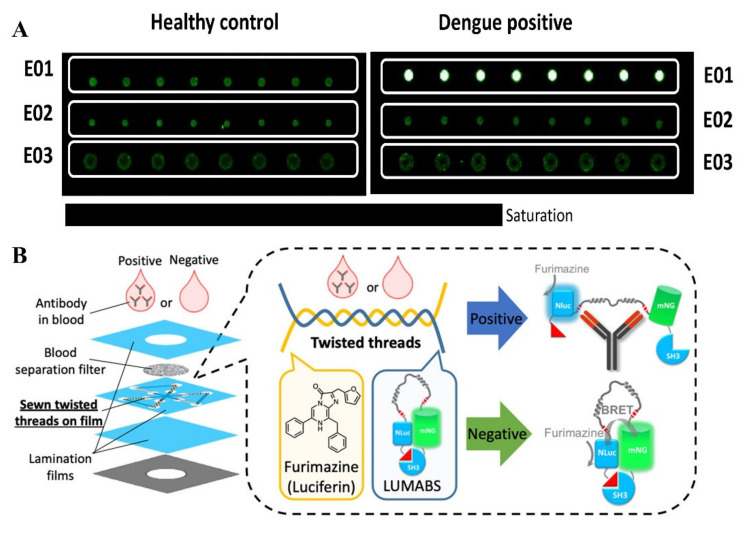
(**A**)Detection of DENV using peptide microarrays. Differentiation of healthy and DENV infection patients’ sera by spotting the fluorescence (reprinted from [200]). (**B**) Detection of anti-DENV-1 antibody from whole blood using an intertwisted thread-based analytical device (Reprinted with permission from ref. [206]. Copyright 2020 American Chemical Society).

Microarrays are sensor-based arrays that can detect multiple target biomarkers simultaneously [197]. The microarrays provide thousands of tiny spots that contain a DNA sequence that acts as a probe to detect gene expression [198]. Usually, mRNA molecules in sample are first converted into cDNA. The cDNA is labeled with a fluorescent probe with differing colors, and the samples are mixed together and allowed to bind in the microarray [199]. This allows parallel testing that is very advantageous in a POC setting to detect the DENV. Bergamaschi et al. developed a synthetic epitope probe based DENV E protein detection assay that mimicked antigenic determinants to efficiently detect DENV and can be implemented as a rapid detection assay [200]. They have synthesized three peptides that have been tested against 20 healthy and 20 DENV patient’s serum to specifically detect IgG of the infected patients (Figure 8A). The sensitivity for detecting E protein was 95% and the assay was 75% specific [200]. Moreover, a DNA microarray was developed by Díaz-Badillo et al. that can distinguish between the dengue serotypes [201]. They showed a high sensitivity that was equivalent to RT-PCR. Microarray provides a high throughput, so that it can be advantageous in that regard. Furthermore, a microarray-based assay was also able to distinguish between dual infections with different stereotypes [201]. Clenton et al. developed a novel microarray using recombinant NS1 proteins to detect various flaviviruses [202]. The multiplex had promising results for DENV, where it can be compared to current commercial ELISA kits. When looking at antibodies from the E protein of the virus, the sensitivity was 91%, while antibodies to the NS1 protein were 99% sensitive. Furthermore, the microarray had a better sensitivity with IgG DENV than with IgM, which can be attributed to sampling timing and prior infections. The IgG sensitivity was 92%, while the specificity was 99%, whereas the IgM sensitivity was 86%, and the specificity was 98%. Recently, a study conducted by Thao et al. has looked into Clenton’s protein microarray and its ability to detect past DENV infections [203]. The researchers used the microarray to test DENV after 1 and 6 months of infection. The study tested 368 serum specimens from 184 infected individuals (16 samples were omitted owing to not having control). Using their binomial model, they were able to have a 92% accuracy on primary past infections but only a 45% accuracy on past secondary infections [203]. Microarrays have many inherent benefits, such as high throughput and high sensitivity. However, they can be very costly and need a person with experience to conduct the test. In some aspects, it could not be used in some rural areas due to its high cost and labor. However, there is potential in the future for its use in POC settings.

Threads can be an alternate mode of liquid transport using capillary wicking principle needing no microchannel fabrication and can be a cheaper alternative to paper-based devices [204]. This emerging technology is based on materials that are readily available, easily reproducible, affordable, and highly portable [205]. Kosuke et al. has developed a microfluidic thread based analytical device which is hydrophobic, intertwisted and sewn inside two lamination films (Figure 8B) [206]. Bioluminescence resonance energy transfer-based technology with the integration of cellphone was utilized to detect multiple antibodies including anti-HIV-1, anti-hemagglutinin or anti-DENV-1 from the spike antibodies in whole blood. The lower limit of detection for anti-DENV-1 was 14.9 nM. This assay can provide both quantitative and qualitative results within 5 min.

## 5. Futuristic CRISPR-Based Assays

**Figure 9:**
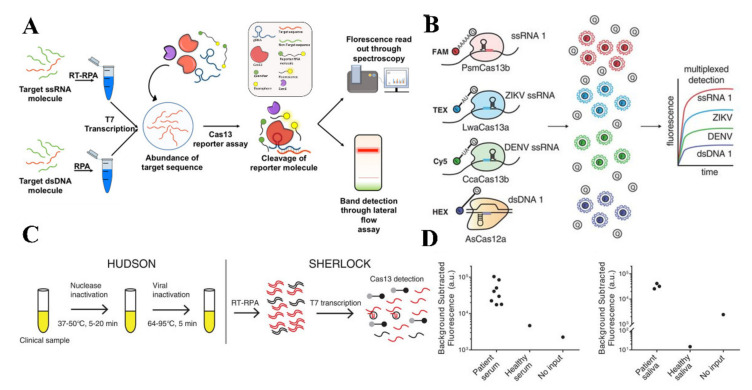
Detection of DENV using CRISPR-Cas based assays. (**A**) Graphical representation of SHERLOCK for detecting nucleic acids (reprinted from [215]). (**B**) Detection of in-sample four-channel multiplexed targets using SHERLOCK version 2 (Reprinted with permission from ref. [216]. Copyright 2018 The American Association for the Advancement of Science.). (**C**,**D**) The detection principle of clinical viral specimens from serum or saliva combining HUDSON and SHERLOCK (Reprinted with permission from ref. [217]. Copyright 2018 The American Association for the Advancement of Science.).

The clustered regularly interspaced short palindromic repeats (CRISPR)-based genome editing technology have become very popular in the past decade due to its flexibility and robustness in diverse applications, including molecular diagnostics, epigenetic modulation, and therapeutics [207,208,209,210]. The CRISPR-Cas system, a simple and powerful nucleic acid detection technology, has numerous advantages, including rapidness, higher sensitivity, and low cost [211]. The CRISPR-Cas system uses “programmable/guided RNA” that detects the viral genomic RNA/DNA (Cas12a and Cas13, programmed to identify DNA or RNA, respectively) and guides the Cas to edit the sequence. This technology encompasses both hybridization and recognition of the target sequence very efficiently, making this technology highly specific and sensitive [212]. Gootenberg et al. has developed a Cas-13 associated specific high-sensitivity enzymatic reporter unlocking (SHERLOCK) platform for detecting infectious diseases with a capability to detect a single-molecule of DNA/RNA [213]. In SHERLOCK, the genomic target sequence is amplified by RPA or RT-RPA method for DNA and RNA sequences, respectively. After T7 transcription of the amplified sequence, the genomic target is detected by Cas 13. Finally, the detection of the target can be done using lateral flow reaction or measuring the fluorescence (Figure 9A) [214,215]. However, in SHERLOCK version 2, a huge advancement has been made by adding a multiplexing detection option, improved sensitivity, and lateral flow readout capability (Figure 9B) [216]. This version is more suitable for POC applications due to the addition of portable lateral flow result readability. This rapid and quantitative detection assay has a sensitivity of 2 aM of DENV. Moreover, the same team made the technology to be field deployable by eliminating the viral DNA/RNA extraction step by Heating Un-extracted Diagnostic Samples to Obligate Nuclease (HUDSON) and then combining with SHERLOCK (Figure 9C,D) [217]. This method can detect DENV directly from patient samples within less than 2 h with a sensitivity of 1 copy/µL. This assay requires minimal expensive equipment making it more inexpensive and suitable for fighting a global crisis.

## 6. Future Direction and Conclusions

DENV infects approximately 400 million people every year, with testing being paramount to diagnosis and treatment for the disease. Some Asian and Latin American countries still face severe illness and deaths due to DENV [9]. Managing the DENV spread to these countries early and rapid diagnostics play an essential role due to the lack of effective treatments. Roughly 66% of the world’s population uses mobile phones, which can be used to educate individuals around the world on prevention [218]. Social media-based platforms can also inform reemergence of outbreaks and get health communication where medical resources are limited [219]. From the diagnostics point of view, the conventional technologies currently being used are mainly applicable to laboratory/medical care-based facilities. All the discussed method of detection for DENV is summarized in Table 1. Mostly these detection assays are costly, requiring sophisticated equipment as well as skilled technician. Though, it is very common that serological assays show cross-reactivity with other flaviviruses. As a result, the development of low-cost, robust, easily deployable, disposable, and accurate diagnostic assays is the key to fight against DENV outbreaks/reemergence in the future.

Asymptomatic and mild cases make it harder to mitigate the spread of the virus. Currently, there are numerous commercial options for individuals, with each having its pros and cons. IgM tests are usually used after 7 days of exposure. The current rapid tests do not meet the standards in terms of sensitivity to be used to diagnose Dengue infection reliably. The NS1 tests allow physicians to test within 1–7 days of infection and can be a cost-effective compared to NAT’s. There is only one FDA approved NS1 test that achieves a sensitivity of 91%. NAT’s can be expensive but can test someone for DENV between 1–7 days of exposure. The current CDC trioplex allows someone to test Zika, Chikungunya, and Dengue. However, further research should be focused on developing new biomarker panels to determine the severity stage of the infection as well as differencing primary and non-primary infections. Evaluating the performance of the state of art bio/nano sensing technologies, including CRISPR, microfluidics, and others, should be established. Smartphone-based assays can also play crucial contribution in the advancement of POC and self-testing assays by excluding the use of expensive laboratory devices. Moreover, a general limitation of various diagnostic platforms is the requirement of extensive sample preparation that limit the use of various assay for POC testing. Efforts should be made to develop innovative solutions that either do not require sample preparation or automate the sample preparation on a single device [176,220].

The future of testing can be paramount in improving rapid tests that are affordable and sensitive. Variation in serotypes and genomic structures is one of the major concerns that need to be considered in the diagnostic development process. Although a single confirmatory test is challenging to develop, it is required for proper patient management. These research directions should be explored to develop single and rapid confirmatory tests in the future. These types of tests can make a difference in rural areas where access to traditional tests cannot be attained. There are also other novel testing technologies under development, such as LODc, LOC, LFA, SPR, microarrays, and EIS. These techniques can cut costs and have the potential to be used in POC settings. On the other hand, hybridization of different technologies such as paper–hydrogel [221], paper-thread [222] based technologies can be used for enhancing the diagnostic sensitivity and reduce costs. The FDA has not cleared any of these emerging products for clinical use yet. Priorities should be given to commercialize more and more POC-based assays as they can significantly improve the disease surveillance, testing, and management.

## Figures and Tables

**Table 1:** Summary of conventional and POC detection methods with their advantages and limitations.

Detection Method	Advantages	Limitations	Target
Serological	Comparatively fast, easier to execute, less expensive	Expensive device required shows cross-reactivity	NS1,IgA, IgG and IgM
PCR	Accurate, early stage detection, muliplexibility, highly sensitive and specific, selective	Only suitable for high resource available settings, skilled personnel needed, prone to contamination, laborious and time consuming	RNA
Isothermal	Fast, no need of thermocycler, simpler than PCR, early stage detection	Less multiplexibility than PCR, prone to primer dimer due to high no of primers.	RNA
SPR	Real time detection, label free, low sample comsumption, early detection capability.	Lower sensitivity, susceptible to nonspecific binding	NS1, IgG and IgM
EIS	Inexpensive, label free, high throughput, sensitive, requires a low volume of samples.	Cumbersome sample preprocessing, requires cleanroom access (sometimes),	RNA, NS1
SERS	Highly specific, simple sample preparation, high throughput	Lacks robustness and reproducibility, highly expensive Raman reader	NS1, DENV Gene
LOC	Disposable, automation potential, cheaper, POC applicable, low reagent consumption, sample-in and answer-out	Manual sample loading, requires an expensive device fabrication process (sometimes),	IgM/IgG, NS1, E, and RNA
LFA	Easy, fast, no sample processing, cheaper than conventional methods.	Mishandling can be occurred, cross-reactivity, qualitative/semi-qualitative result	NS1, IgG, IgM, IgA,
LODc	Disposable, automation potential, cheaper, POC applicable, low reagent consumption	Labor intensive, requires 3D printing access, expensive equipment for device fabrication	RNA
μPAD	Disposable, automation potential, cheaper, POC applicable, low reagent consumption, smartphone integration	Manual sample loading, lower sensitivity, qualitative/semi-qualitative result	NS1, IgM
Microarray	Multiplexity, higher sensitivity, high throughput	Expensive, skilled personnel needed, lengthy time of execution, not POC applicable	Gene expression of DENV
Threads	Disposable, biocompatible, reproducible, cheaper, portable, readily available	Early stage of development, flow manipulation	Anti-DENV antibody
CRISPR	Rapid, highly sensitive, cheaper, simple	non-specific binding,	RNA

## Data Availability

Not Applicable.
